# Methylene blue mitigates lung injury in HCA rats by regulating macrophage pyroptosis via Nrf2/HO-1 and NLRP3 pathways

**DOI:** 10.17305/bb.2025.11851

**Published:** 2025-02-13

**Authors:** Fuyan Ding, Hong Wang, Gang Qiao, Zhidong Zhang

**Affiliations:** 1 Department of Vascular Diseases Intensive Care Unit, Zhengzhou University Central China Fuwai Hospital, People’s Hospital of Zhengzhou University, Henan, China; 2 Department of Vascular Surgery, Zhengzhou University Central China Fuwai Hospital, People’s Hospital of Zhengzhou University, Henan, China

**Keywords:** Methylene blue, MB, hypothermic circulatory arrest, HCA, Nod-like receptor protein 3, NLRP3 inflammasome, nuclear factor erythroid-2 related factor 2 (Nrf2)/heme oxygenase-1 (HO-1) pathway, pyroptosis

## Abstract

Methylene blue (MB) has antioxidant properties, yet its role in acute lung injury (ALI) induced by hypothermic circulatory arrest (HCA) remains unexplored. This study investigates MB’s effects and underlying regulatory mechanisms in an HCA rat model. Rats received an intravenous bolus of MB (1 mg/kg) 15 min before HCA induction. Physiological parameters were monitored, and bronchoalveolar lavage fluid (BALF) was collected 2 h postoperatively to assess total protein levels, inflammatory cells, and cytokines. Histopathological lung damage was evaluated using hematoxylin–eosin (H&E) and TUNEL staining. Inflammatory markers and oxidative stress indicators were measured via ELISA and dihydroethidium (DHE) staining. Alveolar macrophages (AMs) were isolated to analyze polarization using flow cytometry and immunofluorescence double staining. Pyroptosis in AMs was detected with Yo-Pro-1 and Hoechst 33342 staining. Additionally, Western blotting was performed to examine the nuclear factor erythroid-2 related factor 2 (Nrf2)/heme oxygenase-1 (HO-1) pathway, Nod-like receptor protein 3 (NLRP3) inflammasome, and pyroptosis-related proteins. Following HCA, rats exhibited significant blood gas abnormalities, structural lung damage, increased pathological scores, and higher apoptosis rates. However, MB mitigated these effects, improving physiological parameters and reducing lung histopathology scores. MB also lowered proinflammatory cytokine levels, increased SOD and GSH-Px activity, promoted AM polarization toward the M2 phenotype, and decreased pyroptosis. Mechanistically, MB activated the Nrf2/HO-1 pathway while inhibiting NLRP3 inflammasome activation. Notably, Nrf2 inhibitors and NLRP3 agonists weakened MB’s protective effects by promoting inflammasome activation and pyroptosis, whereas Nrf2 agonists and NLRP3 inhibitors enhanced MB’s beneficial impact. In conclusion, MB attenuates HCA-induced ALI by modulating AM polarization and pyroptosis via Nrf2/HO-1 pathway activation and NLRP3 inflammasome inhibition.

## Introduction

Hypothermic circulatory arrest (HCA) is a complex cardiopulmonary bypass (CPB) technique that has become essential for open-heart surgery since its introduction in the 1950s [1, 2]. By lowering tissue metabolism and oxygen consumption, HCA enhances the body’s tolerance to ischemia and hypoxia while also providing a relatively bloodless surgical field, which increases the chances of success [3, 4]. Despite continuous advancements in CPB tubing materials and HCA techniques, many patients still experience postoperative complications of varying severity, including acute lung injury (ALI), neurocognitive dysfunction, and acute kidney injury [5–7]. Notably, the incidence of ALI exceeds 35% [8]. The mechanisms underlying HCA-induced ALI are complex and involve oxidative stress from reactive oxygen species (ROS), systemic inflammatory responses, and lung ischemia/reperfusion injury [9, 10]. Alveolar macrophages (AMs), the primary immune cells in lung tissue, play a crucial role in inflammation, and their death exacerbates ALI progression [11, 12]. Pyroptosis, a form of programmed inflammatory cell death primarily mediated by gasdermin-D (GSDMD), has been implicated in ALI, cardiovascular diseases, and cancer [13–15]. The interaction between AM pyroptosis and inflammation further amplifies lung tissue damage, disrupting alveolar structure and homeostasis, ultimately contributing to ALI onset and progression [16]. The Nod-like receptor protein 3 (NLRP3) inflammasome, which consists of NLRP3, caspase-1, and apoptosis-associated speck-like protein (ASC), is a key component of the innate immune system and plays a central role in metabolic inflammatory responses [17, 18]. Activated caspase-1 generates the N-terminal fragment of GSDMD (GSDMD-N), which ultimately triggers pyroptosis [19]. Therefore, inhibiting AM pyroptosis mediated by the NLRP3 inflammasome is crucial for mitigating HCA-induced ALI.

Methylene blue (MB) is an aromatic compound with a heterocyclic structure that serves various functions in both biology and chemistry, including its use as a dye in the textile industry [20]. Notably, MB has a low redox potential (11 mV), which enables it to catalyze redox reactions and reduce ROS production [20]. Clinically, MB has demonstrated efficacy in treating conditions, such as carbon monoxide poisoning, methemoglobinemia, infectious shock, and urinary tract infections, among others [21–23]. Additionally, MB exhibits anti-inflammatory properties by reducing nitric oxide synthase activity and inhibiting inflammatory signaling, thereby providing neuroprotection [24]. In support of this, microglia exposed to lipopolysaccharide showed a reduction in inflammatory gene expression when MB was introduced to the culture medium [25]. However, no studies have explored the role of MB in NLRP3 inflammasome activation, pyroptosis of AMs, or the associated regulatory mechanisms. Nuclear factor erythroid-2 related factor 2 (Nrf2) is a key modulator of cellular antioxidant responses, regulating the expression of antioxidant, anti-inflammatory, and detoxification-related genes [26]. Under oxidative stress, cytoplasmic Nrf2 dissociates from Keap1 and translocates into the nucleus, where it enhances the expression of heme oxygenase-1 (HO-1) [27]. Activation of the Nrf2/HO-1 pathway has been shown to mitigate inflammation and oxidative stress-related damage in various diseases [28–30]. However, whether MB can alleviate lung injury caused by HCA by activating the Nrf2/HO-1 pathway remains unknown. In this study, we established an HCA-induced ALI rat model to investigate whether MB attenuates ALI by inhibiting the pyroptosis of AMs and its associated regulatory mechanisms. Our findings aim to provide theoretical support for the potential use of MB in ALI management.

## Materials and methods

### Animal feeding

Healthy male SD rats (5 per cage) were obtained from Vital River Laboratory Animal Technology Co., Ltd. (Beijing, China) and housed in a standard animal breeding room for a one-week acclimation period. The room temperature was maintained at 22 ± 2 ^∘^C with a relative humidity of 45%. The rats had free access to food and water. All facilities, including food containers, cages, water bottles, and drinking spouts, were regularly sterilized. All animal experiments were approved by the Zhengzhou University Animal Ethics and Welfare Committee (Approval No. 2022 Ethics (33)) and conducted in accordance with the Code of Ethics for Laboratory Animals.

### Animal grouping and treatment

SD rats were randomly divided into seven groups (*n* ═ 30/group): sham, HCA, HCA+MB, HCA+MB+TBHQ (TB), HCA+MB+ML385 (ML), HCA+MB+Nigericin (Nig), and HCA+MB+MCC950 (MCC). Sham group: Rats underwent anesthesia, tracheal intubation, ventilation, and vascular cannula placement.HCA group: Rats received CPB and HCA treatment. HCA+MB group: Rats received MB (1 mg/kg, M9140, Sigma-Aldrich, St. Louis, MO, USA) via push administration 15 min before HCA induction [31]. HCA+MB+TB group: The Nrf2 agonist TB (20 mg/kg, HY-100489, MedChemExpress, Monmouth Junction, NJ, USA) was administered intraperitoneally 1 h before HCA induction, and MB was given 15 min before HCA induction [31]. HCA+MB+ML group: The Nrf2 inhibitor ML (30 mg/kg, HY-100523, MedChemExpress) was administered intraperitoneally 1 h before HCA induction, followed by MB 15 min prior [32]. HCA+MB+Nig group: The NLRP3 agonist Nigericin (Nig, 1 mg/kg, HY-127019, MedChemExpress) was administered intraperitoneally 1 h before HCA induction, with MB given 15 min prior [32]. HCA+MB+MCC group: The NLRP3 inhibitor MCC950 (MCC, 10 mg/kg, HY-12815, MedChemExpress) was administered intraperitoneally 1 h before HCA induction, with MB given 15 min prior [33].

### Construction of HCA rat model

Referring to the methods of Linardi et al. [34] and Li et al. [35], rats were anesthetized via intraperitoneal injection of sodium pentobarbital (50 mg/kg, P3761, Sigma-Aldrich) and intubated with a 16-gauge cannula before being connected to an animal ventilator. To maintain anesthesia, 1.5% sevoflurane was administered, and mechanical ventilation was set to a tidal volume of 8 mL/kg with a respiratory rate of 60 breaths/min. These parameters were dynamically adjusted based on blood gas analysis results (GEM3500, Radiometer, Copenhagen, Denmark). A pre-heparinized 24-gauge catheter was inserted into the left superficial femoral artery to administer sodium heparin (500 IU/kg) for systemic anticoagulation. Simultaneously, the catheter was connected to a multifunctional physiological monitor (PowerLab, Harvard Apparatus, USA) to measure heart rate and mean arterial pressure (MAP). A venous drain with 24 lateral holes (created using a 12-gauge needle) was inserted into the right external jugular vein to serve as an outlet for venous blood flow. Meanwhile, a 20-gauge catheter was placed in the middle caudal artery to facilitate arterial blood inflow into the CPB circuit. Rectal temperature was continuously monitored using a temperature probe (PowerLab, Harvard Apparatus, USA). Following the method of Kong et al. [9], rats underwent CPB and HCA. Blood drained from the jugular vein entered a venous reservoir before being transported via a rolling blood pump to a membrane oxygenator for gas exchange. After heat exchange, the blood was returned to the body through the caudal artery. Once CPB was established, cooling was initiated using a heat exchanger combined with ice packs for 30 min. When the rectal temperature reached 25 ^∘^C, CPB and ventilation were completely halted, inducing 30 min of circulatory arrest, during which all organs experienced ischemic and hypoxic conditions under hypothermia. At the end of the circulatory arrest period, CPB and mechanical ventilation were restarted. A heating blanket was used to gradually raise the rectal temperature to 35 ^∘^C over 30 min. Once this temperature was reached, CPB and anesthesia were discontinued, and oxygen-enriched mechanical ventilation was maintained for an additional 60 min to allow full recovery from anesthesia. During the resuscitation phase, the remaining perfusion solution was gradually administered to maintain MAP above 80 mmHg. Thirty minutes postoperatively, MAP was measured, and 1 mL of blood was collected from the abdominal aorta for arterial blood gas analysis.

### Determination of total protein concentration

Two hours after CPB surgery, six rats from each group were randomly selected and euthanized via sodium pentobarbital injection (100 mg/kg). Lung tissues were isolated, and bronchoalveolar lavage fluid (BALF) was collected. The right lower lobe of each rat’s lung was removed and placed in a single-use petri dish on ice. The bronchus was identified, secured, and cannulated with a 20 G indwelling needle attached to a 1-mL syringe, then ligated. BALF was obtained by three consecutive lavages with 1 mL of 4 ^∘^C PBS. The collected fluid (about 0.7 mL) was centrifuged, and the supernatant was stored at –80 ^∘^C for further analysis. Total protein concentration in BALF supernatants was measured using a bicinchoninic acid (BCA) Protein Assay Kit (P0012, Beyotime, Shanghai, China).

### Neutrophil and macrophage count

Following the method of Kalidhindi et al. [36], the collected BALF (1 mL) was centrifuged, and the precipitate was resuspended in 1 mL of PBS. The sample was washed again by centrifugation to remove excess washings. A 3-µL smear of the cell sediment was prepared, and Diff-Quik staining solution (G1540, Solarbio, Beijing, China) was added dropwise to cover the smear for 1 min. Macrophages and neutrophils were counted under a light microscope using a haematocrit counter (Z359629, Sigma-Aldrich), with a total of 200 cells analyzed.

### Measurement of lung dry/wet weight ratio

Immediately after the removal of the left upper lung lobe from the rats, the tissue was blotted with filter paper to remove surface fluid. Its wet weight (W) was then measured and recorded. The tissue was subsequently dried at 58 ^∘^C for 48 h, after which the dry weight (D) was recorded. Finally, the W/D ratio was calculated.

### Hematoxylin and eosin (HE) staining

To detect lung tissue lesions in rats, an HE staining kit (C0105S, Beyotime) was used. Lung tissue samples were fixed in 10% neutral formalin solution (F5554, Sigma-Aldrich) for 24 h and then dehydrated in a graded ethanol series (100%, 95%, 75%, and 50%). The samples were embedded in paraffin, sectioned at 4 µm, and baked at 65 ^∘^C for 8 h. The sections were then deparaffinized with xylene (247642, Sigma-Aldrich) and washed with distilled water. Staining was performed using hematoxylin staining solution for 8 min, differentiation solution (C0161S, Beyotime) for 30 s, and eosin staining for 1 min. The sections were sequentially dehydrated in ethanol at different concentrations, cleared with xylene, sealed, and finally examined under an inverted microscope (DM IL LED, Leica, Heidelberg, Germany).

### Histological assessment of lung injury

In each group of rats, histological examination of lung injury was conducted following the methods of Hirao et al. [37] and Lin et al. [38]. The severity of lung injury was assessed by quantifying four key indicators: (I) inflammatory cell infiltration, (II) interstitial edema, (III) alveolar septal congestion, and (IV) alveolar hemorrhage. Each parameter was scored on a scale from 0 to 4, with higher scores indicating more severe injury. The total histopathological score was obtained by summing the four individual scores.

### TUNEL staining

Wax blocks of rat lung tissue were routinely sectioned, deparaffinized with xylene, and dehydrated using a gradient ethanol series. DNase-free proteinase K (20 µg/mL, ST532, Beyotime) was added dropwise, and the samples were incubated for 30 min, followed by three washes with PBS. TUNEL assay solution (C1086, Beyotime) was then added dropwise and incubated for 1.5 h. Subsequently, the samples were stained with DAPI solution (D9542, Sigma-Aldrich) for 10 min in darkness. After sealing with an anti-fade mounting medium (HY-K1042, MedChemExpress), an inverted fluorescence microscope was used for observation and imaging.

### Detection of cellular ultrastructure by transmission electron microscopy

Lung tissues were collected and fixed in 2.5% glutaraldehyde (G6257, Sigma-Aldrich) at 4 ^∘^C overnight. After two rinses with PBS, the samples were treated with 1% osmium tetroxide (O5500, Sigma-Aldrich) for 2 h. Dehydration was carried out using a graded ethanol series (30%, 50%, 70%, 80%, 95%, and 100%) for 10 min at each step, followed by two final dehydration steps with 100% acetone for 10 min each. The samples were then embedded and polymerized at 60 ^∘^C for 48 h. Thin sections (60 nm) were prepared and subjected to double staining with 2% uranyl acetate and lead citrate. After drying, they were mounted on copper grids and observed using a transmission electron microscope (Spectra 200 TEM, Thermo Fisher Scientific), with images recorded accordingly.

### ELISA

Rat lung tissues were cut, thoroughly ground with saline, and centrifuged to collect the supernatant. The levels of superoxide dismutase (SOD, ml077379), malondialdehyde (MDA, ml077384), myeloperoxidase (MPO, ml003250), and glutathione peroxidase (GSH-Px, ml097316) in lung tissue supernatants, as well as intercellular adhesion molecule-1 (ICAM-1, ml003031), interleukin (IL)-6, tumor necrosis factor-alpha (TNF-α, ml002859), and IL-10 (ml002813) in BALF, were assessed using ELISA. All kits were obtained from Enzyme Link Biotechnology (Shanghai, China). Tissue or BALF supernatants were added to the ELISA well plate, followed by the corresponding antibody, and incubated for 1.5 h. After incubation, the antibody solution was discarded, and the wells were washed three times with washing buffer. The washing solution was removed, and the wells were exposed to streptavidin-HRP for 30 min. Substrates A and B were then added and incubated for 10 min. Finally, the termination solution was added and mixed, and the OD450 value was measured immediately using a microplate reader (1410101, Thermo Fisher Scientific, Waltham, MA, USA).

### Determination of ROS levels in lung tissue

Frozen rat lung tissue sections, prepared within 1 h of excision, were treated with a drop of cleaning solution spread evenly across the entire surface and left to stand for 5 min. After aspirating the solution, a 10-µM Dihydroethidium (DHE) probe (S0063, Beyotime) was applied dropwise to ensure uniform coverage and incubated in darkness for 30 min. The sections were then washed twice with PBS, covered with coverslips, and observed under an inverted microscope. Images were captured and analyzed using ImageJ software (version 1.45, Wayne Rasband, National Institute of Mental Health, USA).

### Flow cytometry

The percentage of AM polarization was determined using the method described by Hwang et al. [39]. The obtained BALF was filtered through cell filters, centrifuged, and counted. The resulting cell suspension was added to the Percoll Density Gradient Separation Solution (P4937, Sigma-Aldrich) and subjected to gradient centrifugation according to the reagent instructions. The cell layer at the gradient interface was collected, and the isolated cells were washed with PBS to remove any remaining density gradient separator. The washed cell suspension was then seeded into 6-well plates, and an appropriate amount of DMEM medium (11965092, Gibco, Grand Island, NY, USA) containing 10% fetal bovine serum (F0193, Sigma-Aldrich) was added. Cells were cultured at 37 ^∘^C with 5% CO_2_ for 2–3 h to allow macrophages to adhere. The medium was gently aspirated, and the cells were washed twice with PBS to remove non-adherent cells. Next, 200 µL of a cell suspension containing 1×10^5^ macrophages was taken, and 5 µL of FITC-labeled CD68 primary antibody (11-0689-42, Invitrogen, Carlsbad, CA, USA) and CD163 primary antibody (11-1631-82, Invitrogen) were added separately. The mixture was incubated at 4 ^∘^C for 30 min in the dark. After incubation, 5 mL of sample buffer was added, mixed thoroughly, and centrifuged for 5 min. The precipitate was collected, resuspended in 200 µL of sample buffer, and analyzed using flow cytometry (BD FACSCalibur™ , BD Biosciences, San Jose, CA, USA) to detect fluorescence signals and evaluate the expression of relevant markers for macrophage proportion analysis.

### Immunofluorescence

Following the method of Dai et al. [40], paraffin sections of rat lung tissue were deparaffinized with xylene and dehydrated using a gradient ethanol series. The sections were then placed in 0.1 mol/L citrate buffer (pH ═ 6, S1804, Sigma-Aldrich) for antigen retrieval, microwaved on medium heat for 6 min until reaching a slight boil, and then maintained at medium-low heat for an additional 10 min. The heating was then stopped, and the sections were allowed to cool naturally for 20 min. To permeabilize the tissue sections, 0.3% Triton X-100 (X100, Sigma-Aldrich) was applied for 10 min. Afterward, the sections were blocked with 5% bovine serum albumin (BSA, V900933, Sigma-Aldrich) for 30 min. They were then incubated overnight at 4 ^∘^C with one of the following primary antibodies to evaluate changes in M1 and M2 macrophage proportions: inducible nitric oxide synthase (iNOS) (PA1-036, 1:50, Invitrogen), CD163 (MA5-16656, 1:100, Invitrogen), or CD68 (14-0688-82, 1:50, Invitrogen). To assess the Nrf2/HO-1 pathway, the sections were incubated overnight at 4 ^∘^C with either Nrf2 primary antibody (PA5-27882, 1:100, Invitrogen) or HO-1 primary antibody (PA5-77833, 1:100, Invitrogen). On the following day, FITC-labeled secondary antibody (sheep anti-rabbit IgG, F-2765, 1:100, Invitrogen) was added and incubated for 1 h in darkness. Finally, a DAPI staining solution was applied for 10 min, after which the sections were blocked and observed under a fluorescence microscope.

### Yo-Pro-1 staining and Hoechst 33342 staining

Following the method of Han et al. [41], AMs were collected from each group, and an appropriate amount of Yo-Pro-1 staining solution (200 nM, C1356S, Beyotime) was added to each well, ensuring the dye covered the cell surface. The cells were incubated for 15 min at room temperature, protected from light. Subsequently, nuclei were stained with Hoechst 33342 staining solution (10 µg/mL, C1022, Beyotime) and incubated for 30 min to indicate the total cell number. Under fluorescence microscopy, Yo-Pro-1 produced green fluorescence upon entering pyroptotic cells, while Hoechst 33342 staining resulted in blue fluorescence in the nuclei.

### Western blot

Rat lung tissues and AMs were thoroughly ground, followed by lysis with RIPA buffer (P0013B, Beyotime) to extract proteins. Protein concentrations were quantified using a BCA assay kit. The samples were then subjected to electrophoresis on 12% SDS-PAGE gels (Invitrogen), transferred onto polyvinylidene difluoride (PVDF) membranes (Invitrogen), and blocked with 5% BSA for 3 h. After washing, the membranes were incubated overnight at 4 ^∘^C with the following primary antibodies: Nrf2 (PA5-27882, 1:500, Invitrogen), HO-1 (PA5-77833, 1:1000, Invitrogen), NLRP3 (MA5-23919, 1:100, Invitrogen), pro-caspase-1 (ZRB1233, 1:1000, Sigma-Aldrich), caspase-1 (MA5-16215, 1:200, Invitrogen), ASC (HY-P80548, 1:500, MedChemExpress), GSDMD-N (ab215203, 1:1000, Abcam), or GSDMD (PA5-116815, 1:1000, Invitrogen). The next day, membranes were incubated with a secondary antibody (ab205718, 1:10,000, Abcam) for 2 h. A chemiluminescent substrate (ECL, 34580, Thermo Fisher Scientific) was evenly applied to the membranes, and protein bands were visualized using a gel imaging system (iBright CL1500, Invitrogen). GAPDH (ab263962, 1:1000, Abcam) served as an internal control. Grayscale values of protein bands were quantified using ImageJ software.

### Ethical statement

All rat experiments were approved by Zhengzhou University Animal Ethics and Welfare Committee (approval number: 2022 Ethics No. (33)) and followed the Code of Ethics for Laboratory Animals.

### Statistical analysis

A minimum of three repetitions was performed for each experiment, with results presented as mean ± standard deviation. Statistical analysis was conducted using SPSS 26.0 (IBM SPSS Statistics 26). Multiple group comparisons were performed using one-way analysis of variance (ANOVA). For independent samples, a Student’s *t*-test was used if the data were normally distributed; otherwise, non-parametric tests were applied. A *P* value of < 0.05 was considered statistically significant. Data visualization was carried out using GraphPad Prism 9.0.

## Results

### MB ameliorates HCA-induced abnormalities in physiological indices in rats

**Figure 1.**
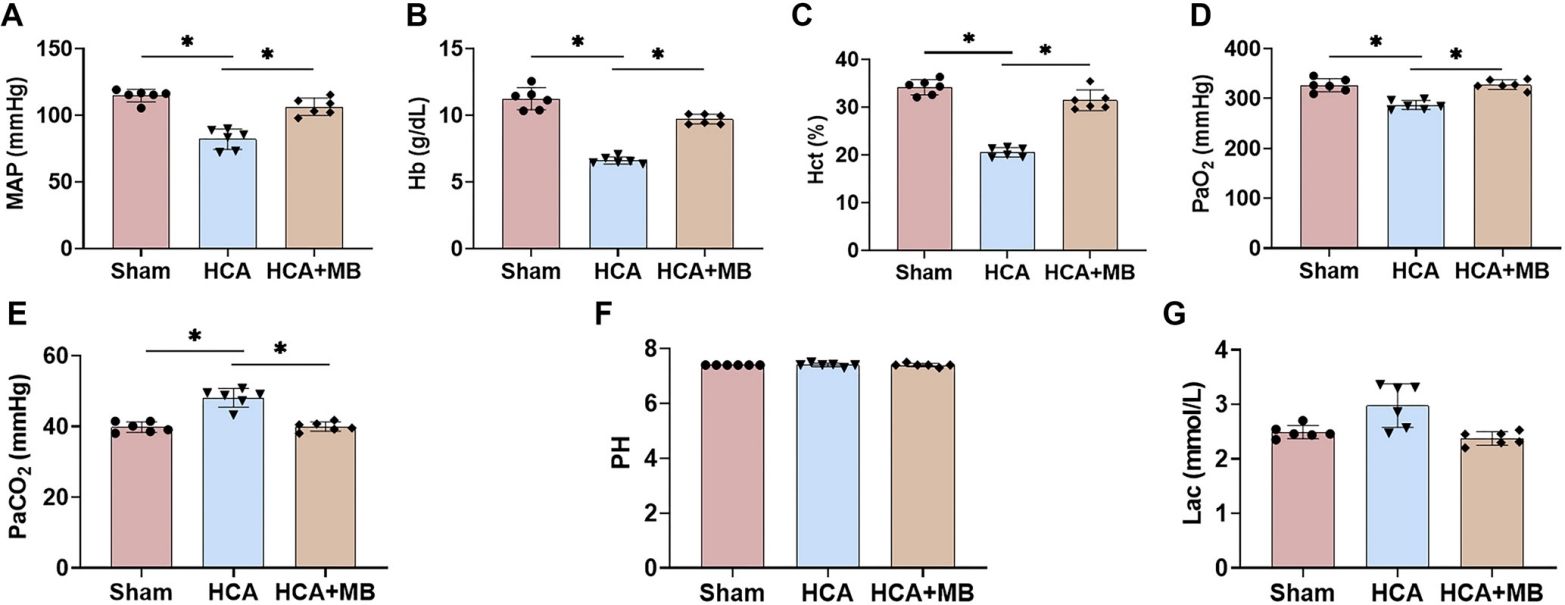
**MB ameliorates HCA-induced abnormalities in physiological indices in rats.** (A) MAP was monitored in rats using a multifunctional physiological monitor 30 min after surgery; Recording of Hb (B), Hct (C), partial pressure of arterial oxygen (PaO_2_), (D) partial pressure of arterial blood carbon dioxide (PaCO_2_) (E), Pondus Hydrogenii (pH) (F), and lactic acid (Lac) (G) by blood gas analyzer. *n* ═ 6, *P < 0.05. MB: Methylene blue; HCA: Hypothermic circulatory arrest; MAP: Mean arterial pressure; Hb: Hemoglobin; Hct: Hematocrit.

At 30 min post-surgery, the MAP of rats was monitored using a multifunctional physiological monitor. Compared to Sham rats, HCA rats exhibited a significant decline in MAP, which was alleviated by MB injection (Figure 1A). Blood gas analysis revealed that both hemoglobin (Hb) and hematocrit (Hct) levels were markedly reduced in HCA rats, likely due to blood dilution from the perfusion of prefilled fluid during the CPB procedure (Figure 1B and 1C). Additionally, after HCA intervention, arterial partial pressure of oxygen (PaO_2_) was notably reduced, while partial pressure of arterial carbon dioxide (PaCO_2_) was significantly elevated. However, MB treatment effectively improved these abnormalities (Figure 1D and 1E). Furthermore, lactic acid accumulation was observed in rats during the 30-min HCA procedure, prompting the use of sodium bicarbonate to correct the resulting acidosis. Notably, MB treatment reduced lactate accumulation (Figure 1F and 1G). These findings confirm the successful implementation of HCA as an interventional process while demonstrating that MB mitigates the physiological abnormalities induced by HCA.

### MB ameliorates HCA-induced histopathological lung injury in rats

**Figure 2.**
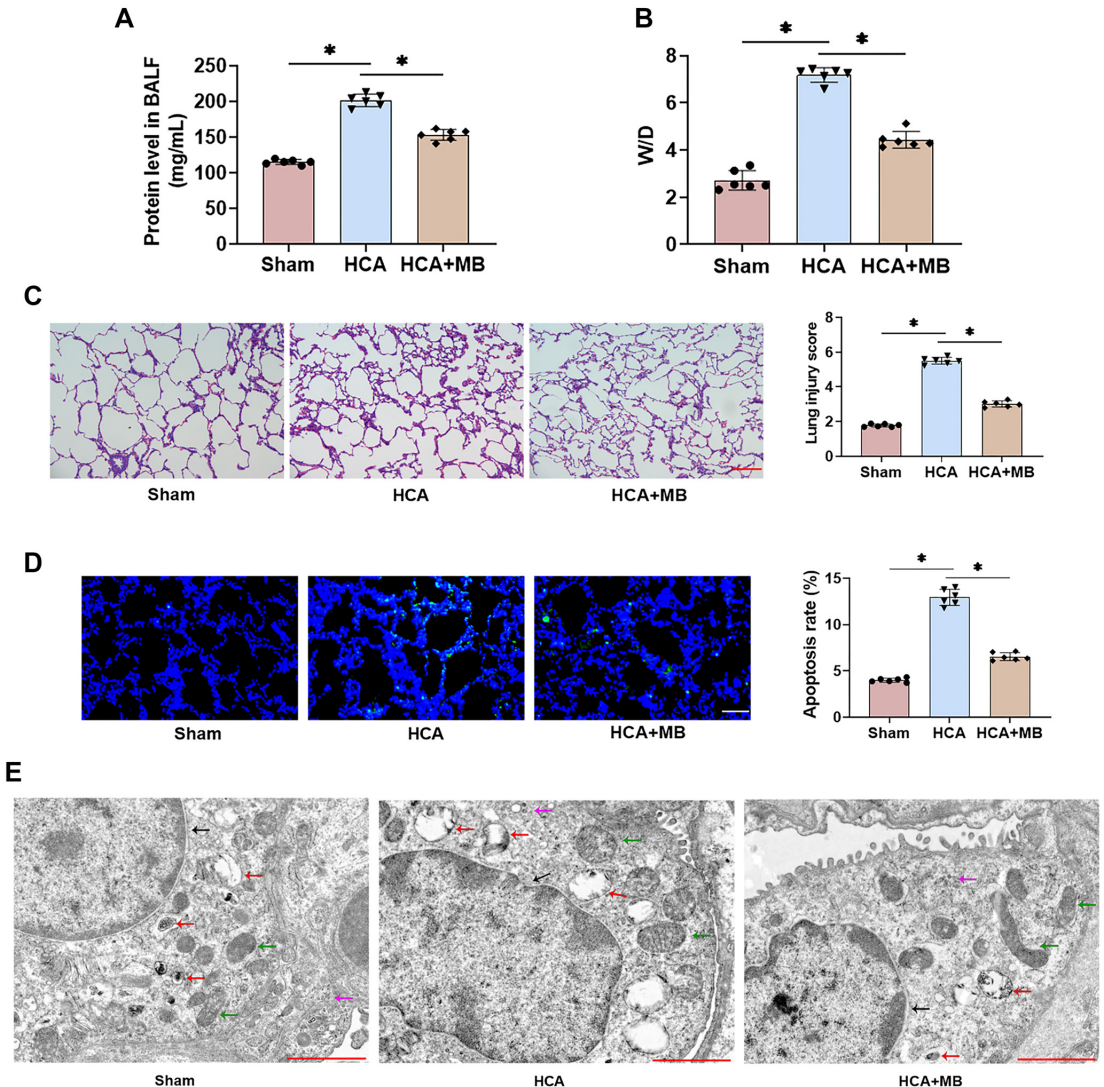
**MB ameliorates HCA-induced histopathological lung injury in rats.** (A) BALF was gathered and total protein concentration in the supernatant was assessed; (B) Calculated the W/D of lung tissue in rats; (C) The pathological changes in rat lung tissue were assessed by HE staining, and scored lung injury (20×, bar ═ 100 µm); (D) TUNEL fluorescence staining to detect apoptosis rate in lung tissues of rats (40×, bar ═ 50 µm); (E) Transmission electron microscopy revealed ultrastructural changes in lung tissue (6k×, bar ═ 2 µm). *n* ═ 6, *P < 0.05. MB: Methylene blue; HCA: Hypothermic circulatory arrest; BALF: Bronchoalveolar lavage fluid; HE: Hematoxylin and eosin.

To assess histological changes in the lungs of rats following HCA, we collected lung tissue samples and prepared BALF 2 h after surgery. HCA intervention led to a significant increase in total protein content and the W/D ratio in rat BALF, whereas MB treatment reduced protein levels and alleviated lung edema (Figure 2A and 2B). HE staining revealed alveolar hemorrhage, alveolar wall thickening, interstitial capillary dilation and congestion, interstitial edema, and a significantly higher pathological damage score in the HCA group (Figure 2C). MB treatment mitigated interstitial capillary dilation and congestion and significantly reduced pathology scores. HCA-induced lung tissue damage was further confirmed by an increase in TUNEL-positive cells and apoptosis rate, both of which were significantly reduced with MB treatment (Figure 2D). Additionally, transmission electron microscopy showed ultrastructural alterations in the alveoli of the HCA group, characterized by small vacuoles in the cytoplasm, mitochondrial edema, and irregular nuclear morphology. In contrast, the HCA+MB group exhibited significantly attenuated ultrastructural changes, with minimal cellular damage, more intact nuclear morphology, homogeneous cytoplasm, and a moderate number of organelles (Figure 2E). These findings indicate that MB significantly improves HCA-induced histopathological changes in the lungs.

### MB ameliorates HCA-induced inflammatory and oxidative stress injury in rat

**Figure 3.**
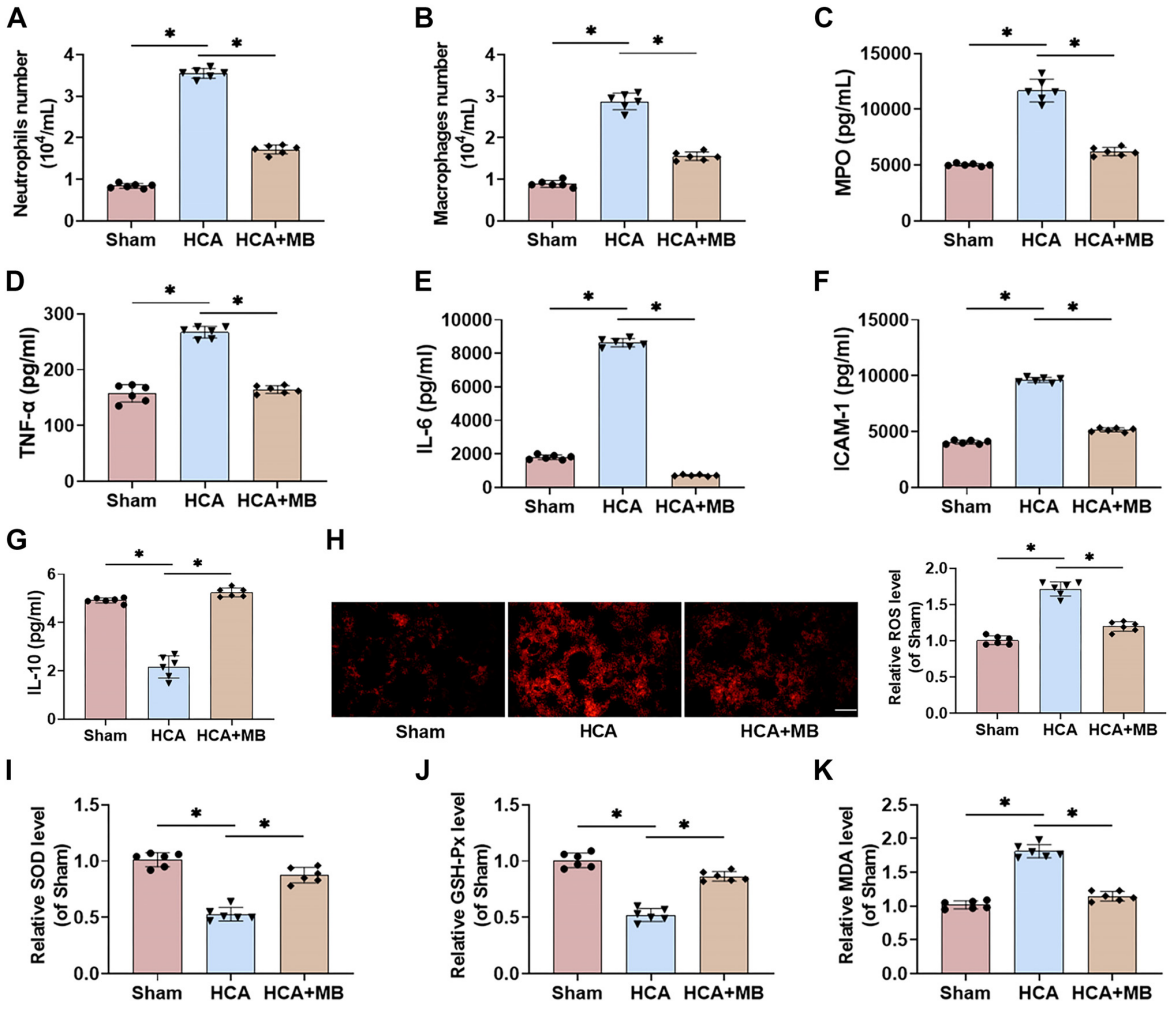
**MB ameliorates HCA-induced inflammatory and oxidative stress injury in rat lungs.** (A and B) BALF was collected and neutrophil and macrophage counts were calculated; (C) Inflammatory factor MPO level in lung tissue was detected through ELISA kit to assess neutrophil infiltration; (D–G) TNF-α, IL-6, ICAM-1, and anti-inflammatory factor IL-10 levels in BALF were assessed by ELISA kits; (H) The ROS level was measured utilizing DHE staining (20×, bar ═ 100 µm); (I–K) To evaluate oxidative stress, SOD, GSH-Px, and MDA levels were assessed by ELISA kits. *n* ═ 6, *P < 0.05. MB: Methylene blue; HCA: Hypothermic circulatory arrest; BALF: Bronchoalveolar lavage fluid; ROS: Reactive oxygen species; IL: Interleukin; DHE: Dihydroethidium.

Following the surgical procedure, the HCA group exhibited a markedly elevated count of macrophages and neutrophils in BALF, indicating increased alveolar wall permeability and a significant inflammatory response in rats. However, MB treatment significantly reduced macrophage and neutrophil counts (Figure 3A and 3B). ELISA findings revealed that MPO levels, a key inflammatory marker, were notably increased in HCA rats, whereas the HCA+MB group showed a significant reduction in MPO levels (Figure 3C). Additionally, the levels of inflammatory factors TNF-α, IL-6, and ICAM-1 were significantly elevated in the BALF of HCA rats, while the anti-inflammatory cytokine IL-10 was markedly reduced. MB treatment effectively mitigated these inflammatory changes (Figure 3D–3G). DHE staining further demonstrated that ROS levels were markedly increased in HCA rats, while MB injection significantly reduced ROS levels (Figure 3H). Moreover, HCA led to a decline in the activity of antioxidant enzymes (SOD and GSH-Px) and an increase in MDA levels, a marker of oxidative stress, in rat lung tissues. MB treatment significantly suppressed HCA-induced oxidative stress (Figure 3I–3K). These findings suggest that MB effectively inhibits HCA-induced lung inflammation and attenuates oxidative stress injury.

### MB promotes polarization of rat AMs toward the M2 type

**Figure 4.**
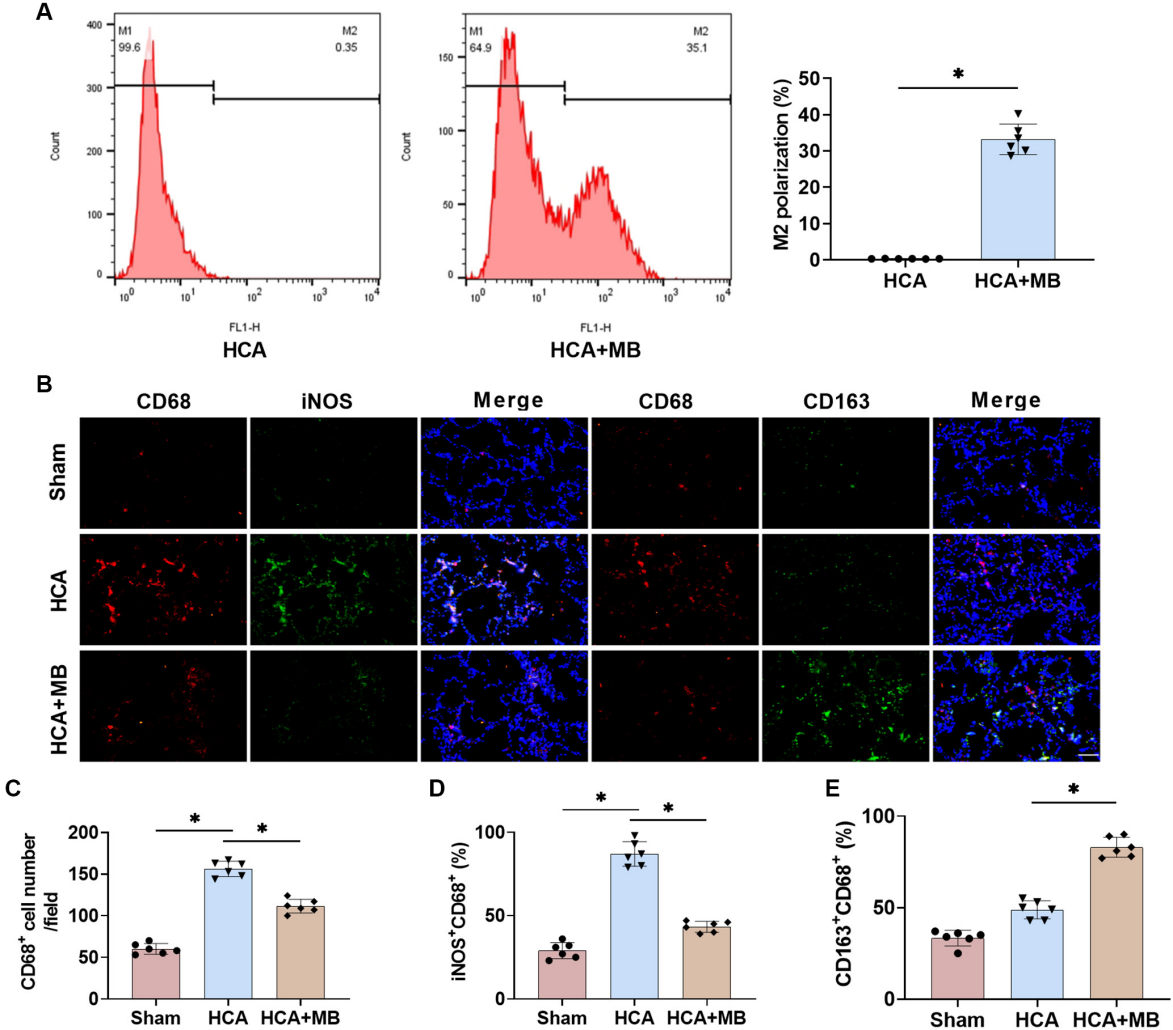
**MB can promote HCA-induced polarization of rat AMs to M2**. (A) The percentage of AMs polarization were evaluated by flow cytometry; (B–E) Immunofluorescence double staining to determine the changes in the percentage of M1 and M2 in rat lung tissue (40×, bar ═ 50 µm). *n* ═ 6, *P < 0.05. MB: Methylene blue; HCA: Hypothermic circulatory arrest; AMs: Alveolar macrophages.

Next, we examined the expression of AM polarization markers after MB treatment to explore its effect on AM polarization. Based on pre-experiment results, we isolated AMs from BALF with a purity exceeding 99%. Flow cytometry results showed that M2-type AMs were nearly absent in the BALF of HCA rats, whereas MB treatment significantly increased their percentage (Figure 4A). Immunofluorescence analysis revealed that HCA rats had a markedly higher number of CD68+ cells and a greater proportion of M1-type (iNOS+CD68+) cells in their lung tissues compared to the Sham group. This suggests that a large population of activated AMs was present in HCA rats, with the vast majority being M1-type (Figure 4B–4E). In contrast, MB treatment significantly reduced the proportion of M1-type AMs in HCA rats and promoted their polarization toward the M2 type, which may be a key mechanism underlying MB’s effects.

### MB activates the Nrf2/HO-1 pathway in HCA rats

**Figure 5.**
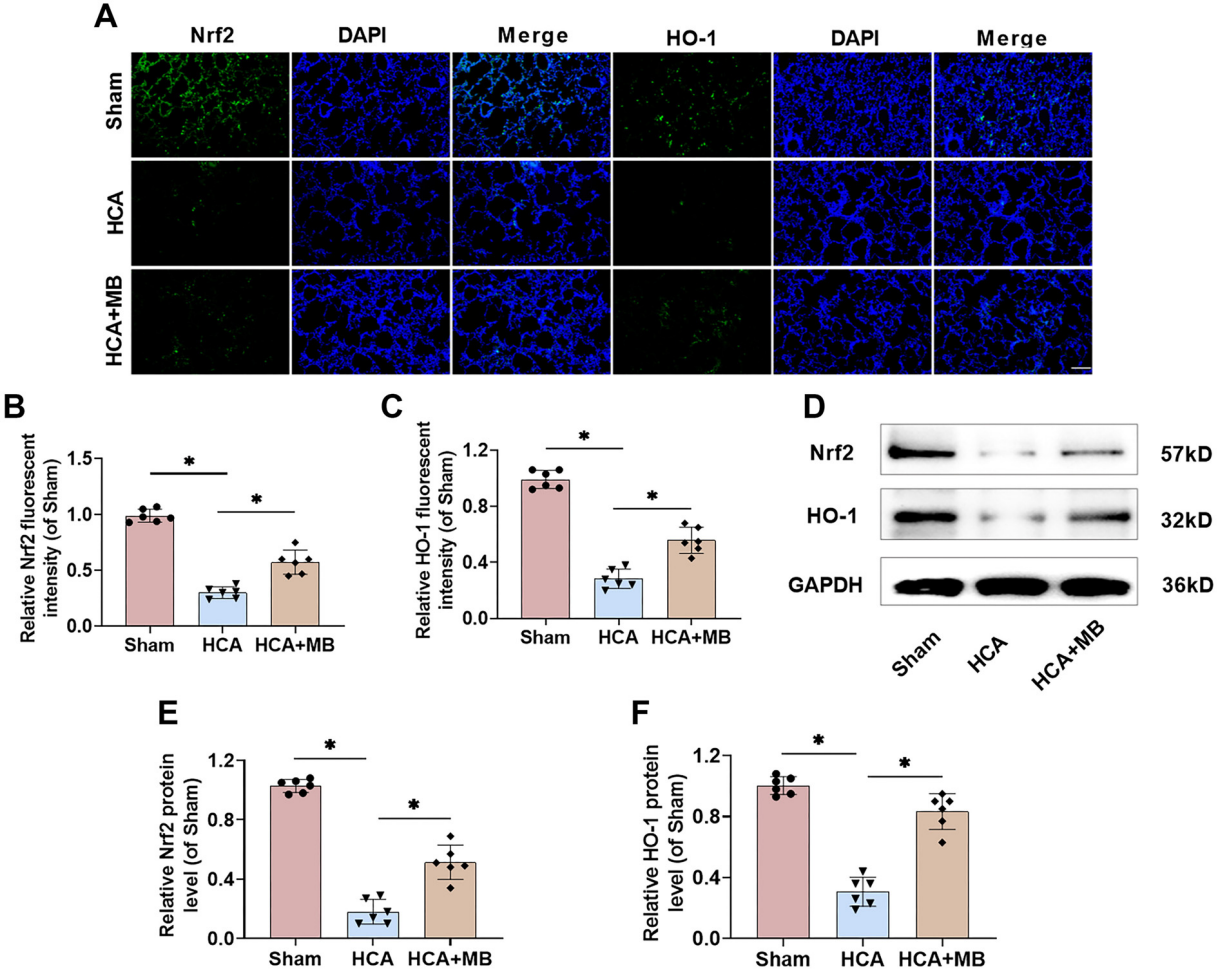
**MB can activate Nrf2/HO-1 pathway in HCA rats.** (A–C) Nrf2 and HO-1 protein levels in lung tissue were assessed by immunofluorescence staining (20×, bar ═ 100 µm); (D–F) Examining Nrf2 and HO-1 levels in lung tissues through Western blot. *n* ═ 6, *P < 0.05. MB: Methylene blue; HCA: Hypothermic circulatory arrest; Nrf2: Nuclear factor erythroid-2 related factor 2; HO-1: Heme oxygenase-1.

Given the crucial role of the Nrf2/HO-1 pathway in inflammation and oxidative stress, we hypothesized that MB’s protective effects on rat lung tissue might be linked to this pathway. Our results showed that HCA significantly reduced Nrf2 and HO-1 levels in rat lung tissues, suggesting that HCA inhibits the Nrf2/HO-1 pathway (Figure 5A–5C). However, lung tissues from the HCA+MB group exhibited higher Nrf2 and HO-1 levels, indicating that MB activates this pathway. Western blot assay results further confirmed this, demonstrating a notable reduction in Nrf2 and HO-1 protein levels in HCA rats, while MB treatment restored these levels (Figure 5D–5F). These findings suggest that MB activates the Nrf2/HO-1 pathway in HCA-exposed rats.

### MB inhibits NLRP3 inflammasome-induced pyroptosis in rat AMs

**Figure 6.**
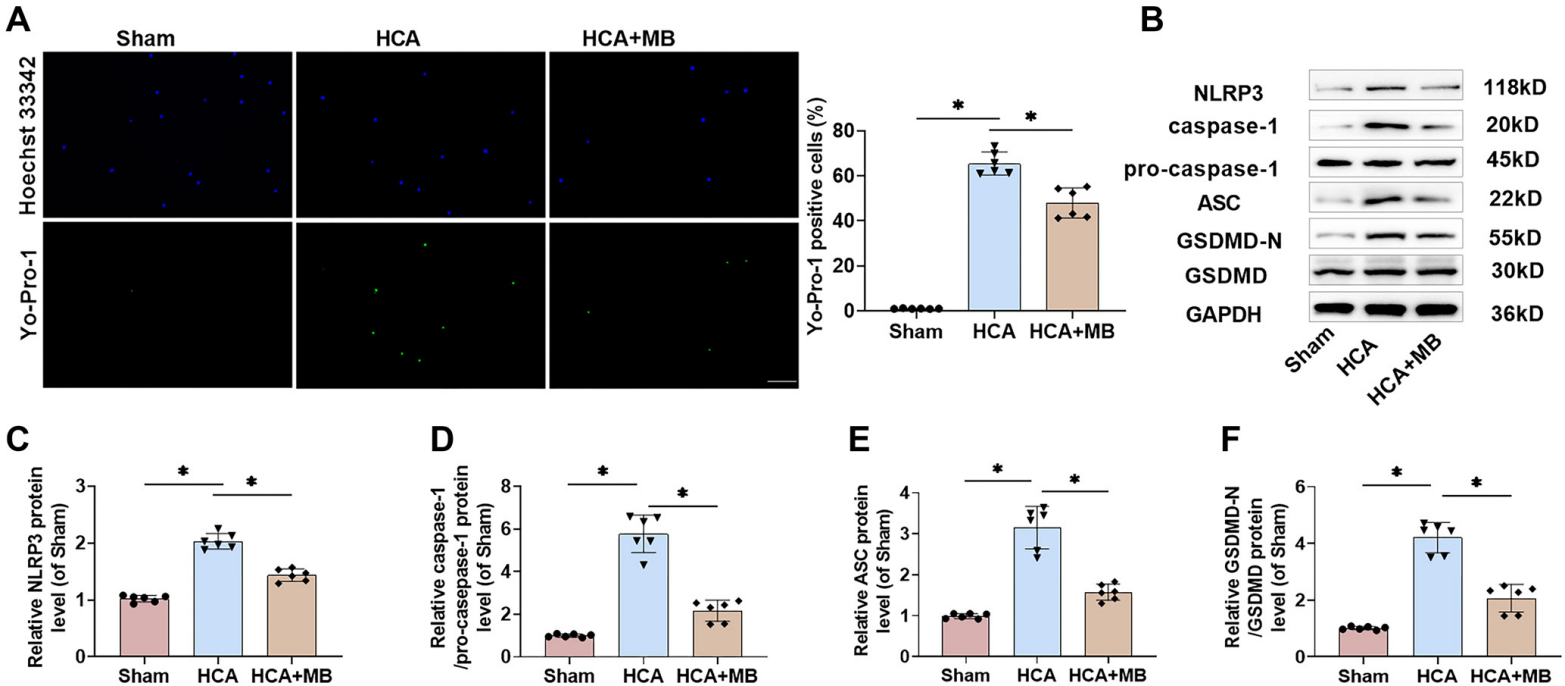
**MB inhibits pyroptosis in AMs caused by NLRP3 inflammasome in HCA rats.** (A) The pyroptosis level of rat AMs was detected by Yo-Pro-1 staining (green) and Hoechst 33342 staining (nuclear blue) (20×, bar ═ 100 µm); (B–F) Western blot bands and relative expression levels of NLRP3, caspase-1, pro-caspase-1, ASC, GSDMD-N, and GSDMD in AMs. *n* ═ 6, *P < 0.05. MB: Methylene blue; HCA: Hypothermic circulatory arrest; AMs: Alveolar macrophages; NLRP3: Nod-like receptor protein 3; GSDMD: Gasdermin-D.

Research indicates that NLRP3 inflammasome activation and cellular pyroptosis are closely linked to the inflammatory pathological process of ALI [42, 43]. Yo-Pro-1 and Hoechst 33342 staining revealed a significant increase in Yo-Pro-1-positive cell counts in the AMs of HCA rats, confirming that HCA induced pyroptosis in these cells. However, MB injection significantly reduced cellular pyroptosis levels (Figure 6A). Additionally, the levels of NLRP3 inflammasome-associated proteins (NLRP3, caspase-1, and ASC) and the pyroptosis-associated protein GSDMD-N were notably elevated in the AMs of HCA rats. In contrast, MB injection markedly suppressed NLRP3 inflammasome activation and pyroptosis (Figure 6B–6F). These findings indicate that HCA triggers NLRP3 inflammasome activation and pyroptosis in rat AMs, while MB effectively inhibits this process.

### ML385 and Nigericin partially revert to MB to inhibit HCA-induced pyroptosis of rat lung macrophages and ameliorate lung injury

**Figure 7.**
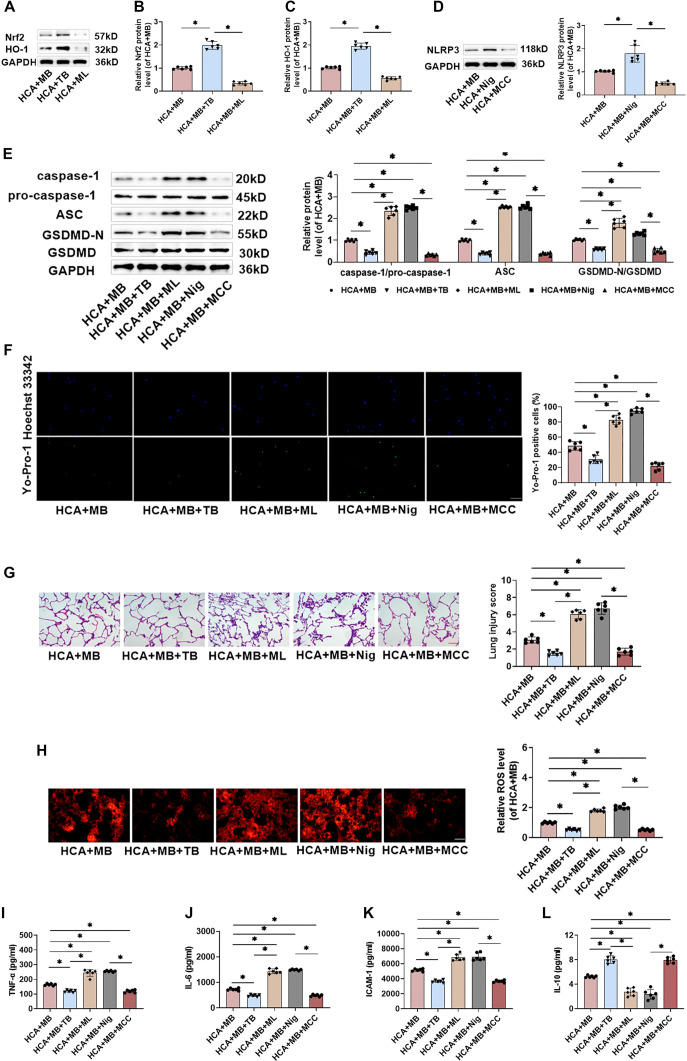
**ML385 and Nigericin can partially restore MB to inhibit HCA-induced pyroptosis of rat lung macrophages and improve lung injury.** (A–E) Western blot bands and relative levels of NLRP3, caspase-1, pro-caspase-1, ASC, GSDMD-N, and GSDMD in Ams; (F) The pyroptosis level of rat AMs was detected by Yo-Pro-1 staining (green) and Hoechst 33342 staining (nuclear blue) (20×, bar ═ 100 µm); (G) Utilizing HE staining to evaluate the pathological changes in rat lung tissue, and to score lung injury (20×, bar ═ 100 µm); (H) The ROS level was measured utilizing DHE staining (20×, bar ═ 100 µm); (I–L) TNF-α, IL-6, ICAM-1, and IL-10 levels in BALF were assessed using ELISA kits. *n* ═ 6, *P < 0.05. MB: Methylene blue; HCA: Hypothermic circulatory arrest; BALF: Bronchoalveolar lavage fluid; AMs: Alveolar macrophages; NLRP3: Nod-like receptor protein 3; ROS: Reactive oxygen species; GSDMD: Gasdermin-D; ASC: Associated speck-like protein; HE: Hematoxylin and eosin; IL: Interleukin; DHE: Dihydroethidium.

To further investigate whether MB exerts a protective effect by inhibiting NLRP3 inflammasome activation and activating the Nrf2/HO-1 pathway, we evaluated the effects of the Nrf2 agonist TB, the Nrf2 inhibitor ML, the NLRP3 agonist Nig, and the NLRP3 inhibitor MCC on MB’s activity. TB injection significantly increased Nrf2 and HO-1 protein levels in HCA+MB rats, whereas ML injection markedly reduced them, confirming that intraperitoneal administration of TB or ML effectively modulates the Nrf2/HO-1 pathway in lung tissues (Figure 7A–7C). Similarly, Nig injection increased NLRP3 levels in HCA+MB rats, whereas MCC injection decreased them, indicating that Nig and MCC effectively regulate NLRP3 inflammasome activation (Figure 7D). Notably, TB injection reduced NLRP3 inflammasome-associated protein levels, GSDMD-N expression, and Yo-Pro-1-positive cell counts in AMs from HCA+MB rats. In contrast, ML injection led to increased protein levels and pyroptotic cell counts, suggesting that MB inhibits NLRP3 inflammasome activation and pyroptosis by stimulating the Nrf2/HO-1 pathway. Similarly, Nig injection promoted NLRP3 inflammasome activation and pyroptosis in AMs from HCA+MB rats, whereas MCC had the opposite effect (Figure 7E and 7F). Furthermore, TB or MCC administration reduced lung histopathological damage, pathological scores, and ROS levels in HCA+MB rats, while ML or Nig exacerbated these effects (Figure 7G and 7H). In the BALF, TNF-α, IL-6, and ICAM-1 levels were significantly reduced, and IL-10 levels were increased after TB or MCC injection, indicating that activating the Nrf2/HO-1 pathway or inhibiting NLRP3 inflammasome activation both alleviated the HCA-induced inflammatory response. Conversely, ML or Nig injection further intensified inflammation (Figure 7I–7L). Overall, these findings demonstrate that MB mitigates HCA-induced histopathological damage, inflammation, and oxidative stress in rat lung tissue by stimulating the Nrf2/HO-1 pathway and inhibiting NLRP3 inflammasome activation.

## Discussion

Our results suggest that HCA surgery induces ALI in rats, leading to lung histopathological damage, inflammatory responses, and oxidative stress injury. Additionally, most AMs following HCA were of the M1 type, and the Nrf2/HO-1 pathway was inhibited in lung tissues, triggering AM pyroptosis and NLRP3 inflammasome activation. MB protected rats from HCA-induced lung damage by reducing inflammation and oxidative stress, promoting the conversion of AMs to the M2 type, activating the Nrf2/HO-1 pathway, and inhibiting both AM pyroptosis and NLRP3 inflammasome activation. Importantly, Nrf2 inhibitors and NLRP3 agonists worsened lung injury, whereas Nrf2 agonists and NLRP3 inhibitors enhanced MB’s protective effects. Therefore, this study is the first to demonstrate that MB can hinder NLRP3 inflammasome activation by activating the Nrf2/HO-1 pathway, thereby mitigating HCA-induced lung histopathological damage, pyroptosis of AMs, oxidative stress, and inflammation. HCA-associated lung injury involves complex physiological and pathological processes, including inflammatory responses, oxidative stress, and ischemia-reperfusion injury [44]. MB acts as an antioxidant that reduces oxidative stress and has potential neuroprotective effects [45, 46]. A cohort study showed that push administration of MB (1–2 mg/kg) improves MAP and reduces norepinephrine levels in patients with extracorporeal circulation postvascular paraplegia syndrome after complex cardiac surgery. Patients with higher MAP responded better to MB, suggesting it should be administered before severe hypotension develops [31]. Additionally, a single intravenous injection of MB (1 mg/kg) 30 min after traumatic brain injury in rats improved motor function [47]. We established an HCA rat model based on the methods of Linardi et al. [34] and Li et al. [35] and administered MB (1 mg/kg) via push injection 15 min before inducing HCA to assess its potential protective effects. MB improved MAP and hematological parameters in HCA rats while alleviating lung tissue damage, suggesting its protective role in lung injury. Moreover, MB has a strong safety profile. A randomized controlled trial found that intravenous infusion of 100-mg MB (dissolved in 500-mL saline) within 24 h of septic shock diagnosis shortened the duration of vasopressor use and ICU stay, with no serious adverse effects reported [21]. Another study assessing MB’s effects and toxicity (0–80 mg/kg) on circulation and metabolism in rats found that doses up to 20 mg/kg had no adverse effects [48]. Taken together, these findings suggest that MB has strong potential for clinical application.

The vascular endothelium serves as the first barrier to lung tissue, preventing inflammatory cells from migrating into the interstitium and alveolar cells. Disruption of endothelial integrity leads to inflammatory cell infiltration and pulmonary edema [49]. Pulmonary ischemia-reperfusion during CPB has been shown to trigger excessive ROS production, activate the monocyte/macrophage system, and stimulate the release of large quantities of inflammatory cytokines and chemokines (e.g., ICAM-1, TNF-α, and IL-6), further promoting neutrophil recruitment into lung tissue [50]. Notably, Abreu et al. [51] demonstrated that MB mitigates lung inflammation and apoptosis caused by non-ischemic ischemia-reperfusion injury following unilateral lung transplantation in rats. In our study, MB effectively reduced inflammatory infiltration in lung tissues of HCA rats, alleviated alveolar epithelial cell injury and pulmonary edema, and enhanced antioxidant enzyme activity. These findings suggest that MB attenuates lung inflammation and oxidative stress injury caused by HCA, consistent with the study by Abreu et al. [51]. A previous study reported that intravenous administration of 9 mg/kg of chlorogenic acid 20 min before modeling in deep HCA rats ameliorated brain tissue injury and reduced plasma TNF-α levels from 15 pg/mL to approximately 7 pg/mL [52]. In our study, administration of 1 mg/kg of MB reduced TNF-α levels in BALF from 267 pg/mL to approximately 165 pg/mL. However, due to differences in animal models and assays, direct comparison is not feasible. Importantly, it has been shown that an infusion of 3 mg/kg of MB alleviates oleic acid-induced ALI and reduces pulmonary edema in rats [53], further confirming MB’s protective effect against lung injury. Macrophage polarization is a process in which macrophages adapt their phenotype to restore homeostasis and enhance their ability to respond to environmental changes [54]. Classically activated M1 macrophages express markers, such as CD80, iNOS, and CD86 and exhibit proinflammatory properties, while selectively activated M2 macrophages express markers, such as CD163, CD64, and IL-10, and possess anti-inflammatory capabilities [55]. During the acute phase of ALI, AMs predominantly polarize toward the M1 phenotype, releasing large amounts of inflammatory factors that exacerbate the inflammatory response [56]. Our findings support this, as the majority of AMs in the BALF of HCA rats were of the M1 type, with almost no M2 macrophages detected. However, MB treatment significantly increased the proportion of M2-type AMs, suggesting that MB may reduce inflammatory lung injury by promoting AM polarization toward the M2 phenotype.

Cellular pyroptosis is a Caspase-1 or Caspase-11/4/5-dependent form of cell death, primarily characterized by the release of inflammatory mediators, cytoplasmic membrane pore formation, and cell swelling and rupture [57]. This process typically begins with the activation of pathogen recognition receptors (e.g., NLRP3), leading to inflammasome formation. Subsequently, pro-Caspase-1 undergoes autohydrolysis to form mature Caspase-1, which induces membrane pore formation and inflammatory mediator release, ultimately triggering an inflammatory response [58, 59]. It has been reported that blood contact with abiotic surfaces during HCA stimulates the release of inflammatory mediators, activates the NLRP3 inflammasome and Caspase-1, and induces alveolar cell pyroptosis [60]. Additionally, the NLRP3 inflammasome has been implicated in oxygen–glucose deprivation and reoxygenation injury associated with HCA [61]. Thus, inhibiting pyroptosis-related molecules (e.g., NLRP3, Caspase-1) could effectively reduce pyroptosis and inflammation triggered by CPB and HCA. In our study, HCA led to NLRP3 inflammasome activation and cellular pyroptosis in rat AMs, whereas MB treatment inhibited this effect. This inhibition may be a key mechanism underlying MB’s lung-protective effects. Several studies have shown that activation of the Nrf2/HO-1 pathway slows the progression of inflammatory diseases and protects lung tissue from injury [30, 62]. For example, Jawad et al. [63] found that activation of this pathway in renal tissue attenuated ischemia-reperfusion injury induced by asphyxial cardiac arrest in rats. Similarly, Luo et al. [64] demonstrated that the Keap1-Nrf2/HO-1 pathway regulates lipopolysaccharide-induced ALI, and its activation reduces inflammation and ferroptosis in lung tissue. While the pathogenesis of HCA-induced ALI remains unclear, previous studies have implicated the Nrf2 pathway in HCA-induced acute kidney injury [35]. In our research, HCA significantly reduced Nrf2/HO-1 pathway-associated protein levels in rats, supporting its involvement in HCA-induced ALI and aligning with earlier findings [64]. Notably, activation of the Nrf2/HO-1 pathway has also been shown to inhibit NLRP3 inflammasome activation and promote M2-type microglia polarization [65]. Given the importance of the Nrf2/HO-1 pathway, we hypothesized that MB exerts a protective effect against lung damage in HCA rats by activating this pathway. Our findings confirmed this hypothesis, as MB treatment increased Nrf2 and HO-1 levels, demonstrating its ability to activate the Nrf2/HO-1 pathway. Furthermore, Nrf2 inhibitors and NLRP3 agonists exacerbated pyroptosis and inflammasome activation while diminishing MB’s protective effects. Conversely, Nrf2 agonists and NLRP3 inhibitors enhanced MB’s effects. These results confirm that MB inhibits cellular pyroptosis by activating the Nrf2/HO-1 pathway and suppressing NLRP3 inflammasome activation, thereby reducing inflammation and oxidative stress-induced lung injury in HCA. Previous research has shown that MB protects vascular neurons by activating the PI3K-Akt pathway and ameliorates cognitive deficits caused by chronic cerebral hypoperfusion [66]. Additionally, MB reduces neuroinflammation after subarachnoid hemorrhage via the Akt/GSK-3β/MEF2D signaling pathway [67]. However, whether these signaling pathways play a role in HCA-induced ALI remains unknown. Future studies should explore whether MB improves HCA-induced ALI through the modulation of these pathways.

## Conclusion

In summary, MB exerts a protective effect against lung damage in HCA rats by promoting the polarization of AMs to the M2 type and reducing cellular pyroptosis. Its mechanism of action appears to be linked to the activation of the Nrf2/HO-1 pathway and the suppression of the NLRP3 inflammasome. This study elucidates the potential mechanism through which MB ameliorates lung injury in HCA rats, expands our understanding of the molecular processes underlying CPB-HCA-induced ALI, and provides a theoretical foundation and data support for the use of MB in treating CPB-HCA-induced ALI. However, there are some limitations to our study that should be noted. First, we did not investigate the dose-dependent effects of MB. Although our chosen doses were based on previously reported data, different doses might yield significantly different outcomes. Future studies will explore the dose-response relationship of MB in greater detail. Additionally, in clinical settings, patients undergoing CPB surgery often have comorbidities, such as hypertension, diabetes, and other systemic dysfunctions. To enhance the clinical relevance of our findings, future research should incorporate more complex experimental designs, including HCA models in hypertensive or diabetic animals.

## Supplemental data

**Highlights:**

1. Methylene blue (MB) ameliorates hypothermic circulatory arrest (HCA)-induced lung-related injury in rats.

2. MB promotes HCA-induced polarization of rat alveolar macrophages (AMs) to the M2 type.

3. MB activates the Nrf2/HO-1 pathway in HCA rats.

4. MB inhibits HCA-induced NLRP3 inflammasome-mediated pyroptosis in rat lung tissue AMs.


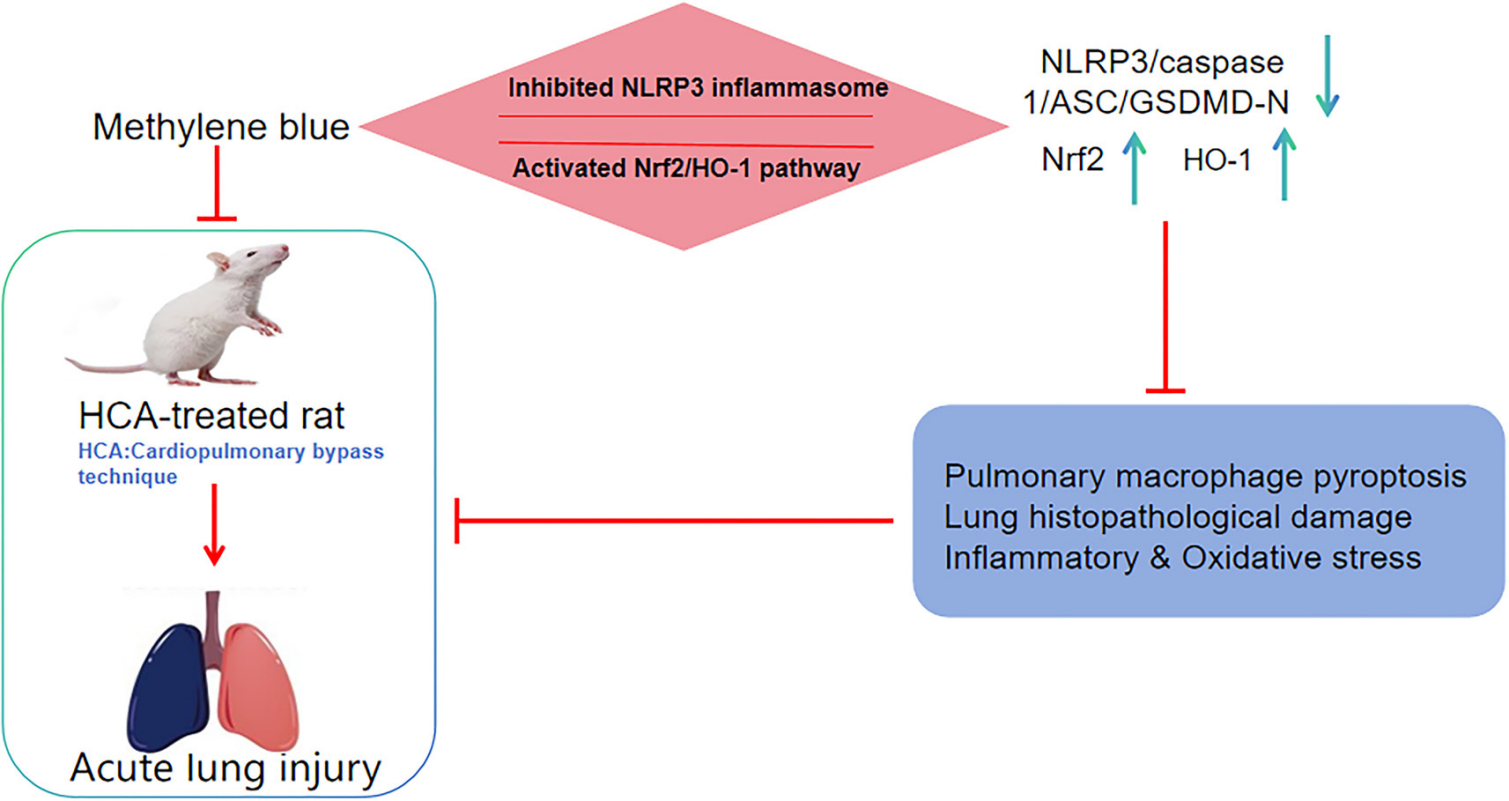


**Graphical abstract:** MB activated the Nrf2/HO-1 pathway and inhibited NLRP3 inflammasome activation, promoted the polarization of AMs toward the M2 type, and alleviated cell pyroptosis, ameliorated the pathological damage of lung tissues, inhibited inflammatory responses and oxidative stress, and thus exerted a protective effect against lung injury in HCA rats.

## Data Availability

To acquire the data that underpins the results of this study, please contact the corresponding author, [TBF].
